# Sclerosing Extramedullary Hematopoietic Tumor

**DOI:** 10.4274/tjh.2017.0438

**Published:** 2018-08-05

**Authors:** Kemal Deniz, Güven Kahriman, İsmail Koçyiğit, Turhan Ökten, Ali Ünal

**Affiliations:** 1 Erciyes University Faculty of Medicine, Department of Pathology, Kayseri, Turkey; 2 Erciyes University Faculty of Medicine, Department of Radiodiagnostics, Kayseri, Turkey; 3 Erciyes University Faculty of Medicine, Department of Nephrology, Kayseri, Turkey; 4 Erciyes University Faculty of Medicine, Department of Hematology, Kayseri, Turkey

**Keywords:** Sclerosing extramedullary hematopoietic tumor, Myelofibrosis, Kidney

## To the Editor,

Sclerosing extramedullary hematopoietic tumor (SEMHT) is a very rare disease. It was first described by Remstein et al. [1] in 2000 and few cases have been reported since then. It is associated with chronic myeloproliferative disorders. We describe a patient with SEMHT with bilateral renal involvement.

**Figure 1:**
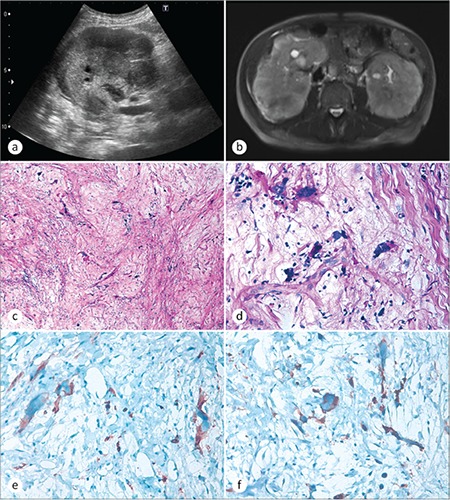
a) Abdominal ultrasonographic image and b) magnetic resonance image showing heterogeneous renal capsular mass. c) Photomicrograph showing large atypical megakaryocytes in a myxoid to collagenous background (hematoxylin and eosin, 100^x^). d) Megakaryocytes at high power view (hematoxylin and eosin, 400^x^), e) showing factor-8 positivity (400^x^) and f) CD41 positivity (400^x^).

A 72-year-old man was admitted to the emergency clinic with dyspnea and abdominal pain. Physical examination revealed decreased breath sound over the right and left lower lung areas and severe edema in both lower extremities. He was clinically overhydrated. The rest of the physical examination was normal. Routine laboratory tests showed the following: serum levels of hemoglobin, 9.8 g/dL; white blood cell count, 31x10^9^/L; platelet count, 100x10^9^/L; blood urea nitrogen, 36 mg/dL; serum creatinine, 1.9 mg/dL; serum albumin, 2.2 g/dL. The peripheral blood smear was normal. A bone marrow biopsy was not performed. Abdominal ultrasound showed a perirenal hypoechoic mass compressing the bilateral kidneys (Figure 1a). Magnetic resonance imaging revealed heterogeneous renal capsular involvement with maximum thickness of 30 mm (Figure 1b). Renal Tru-cut biopsies were performed from both kidneys. Microscopically, both lesions showed myxoid and sclerotic stroma intermixed with large atypical cells (Figures 1c and 1d). Immunohistochemistry revealed positivity for factor-8 (Figure 1e) and CD41 in atypical cells (Figure 1f). Scattered mature myeloid cells were positive for myeloperoxidase, while CD34, CD117, S100, CD3, CD30, CD20, glycophorin, MDM2, keratin, EMA, desmin, and myogenin were all negative. The presence of CD41-positive atypical megakaryocytes within the tumor suggested the diagnosis of SEMHT. His previous history of primary myelofibrosis and splenectomy was learned after the histologic diagnosis of the renal tumor.

SEMHT is an uncommon lesion formerly known as fibrous hematopoietic tumor or myelosclerosis [1]. It is associated with chronic myeloproliferative disorders, mainly chronic idiopathic myelofibrosis in older age groups. SEMHT has a predilection for the mesentery and retroperitoneum. However, tumors involving the skin, liver, kidneys, and lacrimal glands were described as single reports [1,2,3]. SEMHT has usually presented as multiple nodules with varying sizes. Our case showed the diffuse infiltrative nature of the tumor, compressing the bilateral kidneys. Bilateral renal involvement is an unreported radiologic finding. Microscopically, these tumors were characterized by myxoid to sclerotic stroma with thick collagen bundles, intermixed with large atypical megakaryocytes. Occasional foci of mature hematopoietic cells were encountered [1,2]. It is believed that sclerosis within the tumor was produced by fibroblasts, induced by cytokines released from clonal megakaryocytes. The presence of the *JAK2 V617F* mutation may also suggest the clonal nature of the lesion [4].

The differential diagnosis includes sclerosing liposarcoma, malignant fibrous histiocytoma/pleomorphic sarcoma, sarcomatoid/anaplastic carcinoma, and Hodgkin lymphoma [1,3]. The presence of dysplastic megakaryocytes with “ink blot-like” nuclei and eosinophilic cytoplasm is in favor of SEMHT. Factor-8, CD41, and CD61 are helpful markers for the confirmation of the diagnosis. Pathologists should keep this rare entity in mind for the differential diagnosis of tumors with anaplastic morphology. High cellular pleomorphism may lead to inaccurate diagnosis of sarcoma or carcinoma and a subsequent unnecessary surgery.
